# Milk Fat Globule Membrane-Containing Protein Powder Promotes Fitness in *Caenorhabditis elegans*

**DOI:** 10.3390/nu16142290

**Published:** 2024-07-17

**Authors:** Miina Pitkänen, Olli Matilainen

**Affiliations:** The Molecular and Integrative Biosciences Research Programme, Faculty of Biological and Environmental Sciences, University of Helsinki, 00790 Helsinki, Finland; miina.pitkanen@helsinki.fi

**Keywords:** milk fat globule membrane (MFGM), hydrolyzed milk protein, MFGM-containing protein powder (MProPow), *C. elegans*, fitness, innate immunity, cathepsin B

## Abstract

Milk-derived peptides and milk fat globule membrane (MFGM) have gained interest as health-promoting food ingredients. However, the mechanisms by which these nutraceuticals modulate the function of biological systems often remain unclear. We utilized *Caenorhabditis elegans* to elucidate how MFGM-containing protein powder (MProPow), previously used in a clinical trial, affect the physiology of this model organism. Our results demonstrate that MProPow does not affect lifespan but promotes the fitness of the animals. Surprisingly, gene expression analysis revealed that MProPow decreases the expression of genes functioning on innate immunity, which also translates into reduced survival on pathogenic bacteria. One of the innate immunity-associated genes showing reduced expression upon MProPow supplementation is *cpr-3*, the homolog of human cathepsin B. Interestingly, knockdown of *cpr-3* enhances fitness, but not in MProPow-treated animals, suggesting that MProPow contributes to fitness by downregulating the expression of this gene. In summary, this research highlights the value of *C. elegans* in testing the biological activity of food supplements and nutraceuticals. Furthermore, this study should encourage investigations into whether milk-derived peptides and MFGM mediate their beneficial effects through the modulation of cathepsin B expression in humans.

## 1. Introduction

In addition to exercise, nutrition plays a central role in maintaining health and fitness, which is crucial for both societal welfare and individual well-being. Therefore, food supplements and nutraceuticals have become increasingly popular for complementing the diet. These products have the potential to fill nutritional gaps and thereby prevent chronic diseases by providing essential vitamins, minerals, and other bioactive compounds [1]. Milk-derived bioactive peptides, which are naturally present in milk or can be generated through the hydrolysis of native proteins, have garnered attention due to their diverse health benefits and potential applications in functional foods, dietary supplements, and pharmaceuticals. For example, these peptides have been shown to promote cardiovascular health, possess immunomodulatory properties, and improve metabolic health [2,3]. In addition to milk-derived peptides, the milk fat globule membrane (MFGM) is another bioactive component found in milk. MFGM, which encloses milk fat in globules, has a complex trilayer structure mainly composed of polar lipids, cholesterol, various proteins, and glycoproteins [4]. MFGM is recognized for its potential health benefits, especially in infants, as it promotes the maturation of the gut and the immune system and boosts cognitive development [5,6]. Studies conducted on older subjects have found that MFGM-enriched milk improves episodic memory [7], while MFGM supplementation combined with exercise may be beneficial in improving walking speed and other walking parameters, such as step length [8].

Recently, a study reported how protein powder (with partially hydrolyzed protein) and a daily snack rich in both milk-derived peptides and MFGM affect the physical performance of older women [9]. This study found that these dietary interventions improved physical performance based on the Short Physical Performance Battery score [9]. Building on this study, we utilized the widely used model organism, *Caenorhabditis elegans*, to further investigate the effects of MFGM-containing protein powders on a multicellular organism. Due to its small size, ease of culture, and short life span, *C. elegans* is an excellent model to study how different interventions affect an organism’s physiology throughout its life cycle. Notably, despite being a 1 mm long nematode, at least 83% of the *C. elegans* proteome has human homologous genes [10]. Moreover, as *C. elegans* conserves the key signaling pathways regulating health and lifespan across eukaryotes, it is an emerging model in food and nutrition research, as well as in drug discovery [11,12]. By using this model organism, our aim was to investigate whether MFGM-containing protein powders have an effect on fitness or lifespan, and if so, elucidate the underlying mechanism.

## 2. Materials and Methods

### 2.1. MFGM-Containing Protein Powder (MProPow)

This study used two MFGM-containing protein powders (MProPows: MProPow1 and MproPow2), which were made with the same recipe but are from two independent batches. The production of MProPow has been described earlier [9]. Shortly, lactose-free, protein-hydrolyzed butter milk powder was produced from ultrafiltered lactose-free buttermilk concentrate. A part of monosaccharides was removed by ultrafiltration, and proteins and residual fats were concentrated. After ultrafiltration, proteins were partially hydrolyzed by enzymatic hydrolysis. The lactose content of the final protein concentrate was <0.01%. Protein hydrolysis was undertaken according to patent EP 2632277B1 [13] and as described previously [14]. As stated previously, the degree of hydrolysis of the proteins was controlled so that the ratio of free tyrosine to total protein was 6.3 mg per gram, respectively [14]. Nutritional content of MProPow is listed in Table 1. For experiments in this study, MProPows were mixed with water to achieve a final concentration of 1 mg/mL. Notably, MProPows are not fully soluble in water. Therefore, to increase their solubility, they were ground with a plastic pestle in 500 µL of water in a 1.5 mL Eppendorf tube before diluting them to a concentration of 1 mg/mL. MProPow solutions were spread on agar plates (200 µL on a 3 cm plate, 1 mL on a 6 cm plate, and 2 mL on a 10 cm plate). Water was used as a control in all experiments. After the plates dried, they were seeded with bacteria.

### 2.2. C. elegans Maintenance

The *C. elegans* N2 (Bristol) strain was used in all experiments. *C. elegans* were maintained on NGM plates (peptone, P4963, Merck, Darmstadt, Germany); agar, A4550, Merck; NaCl, 746398, Merck). Except for PA14 assay and *cpr-3* RNAi experiments, animals were kept on *E. coli* OP50 or *E. coli* HT115 carrying the empty vector (EV, control vector for RNAi). When using the HT115 (EV), bacterial culture was prepared according to the RNAi protocol described earlier (including the supplementation of IPTG) [15]. Unless otherwise mentioned, all experiments were performed at 20 °C.

### 2.3. Lifespan Analyses

Lifespan experiments were performed on *E. coli* OP50 or *E. coli* HT115 carrying an empty vector (EV). Lifespan experiments were initiated by allowing gravid hermaphrodites (P0 generation) to lay eggs on 6 cm NGM agar plates, and the F1 generation was scored for lifespan. Alternatively, animals were bleached and allowed to hatch overnight in M9 before plating L1 larvae onto 6 cm NGM agar plates. These two alternative methods to initiate lifespan did not affect the conclusions drawn from the experiments. At the L4 larval stage, animals were transferred to 3 cm NGM agar plates containing 5-Fluorouracil (5-FU) (10 µM) (Merck, #F6627) to prevent progeny production. Animals with an exploded vulva or that crawled off the plate were censored. Animals were counted as dead if there was no movement after being poked with a platinum wire. Lifespans were checked every 1–3 days. Animals were maintained on MProPow-containing plates throughout the entire experiment. Mean lifespan ± standard error (SE) is reported in the Supplementary Materials File S1, Table S1.

### 2.4. Motility Measurement

Animals were synchronized by bleaching and plated as L1 larvae on 10 cm NGM agar plates seeded with *E. coli* OP50 or *E. coli* HT115 (EV), which were kept at 20 °C. At the L4 larval stage, animals were transferred to 10 cm NGM agar plates containing 5-FU (10 µM) (Merck, #F6627) to prevent progeny production. Motility was measured on days 2 and 4 of adulthood. Animals were transferred to new plates after day 2 of adulthood. For motility measurement, 10 animals were picked in a single well of a 96-well plate containing 100 µL of M9 solution. In a single experiment, 22–24 wells were used for each condition. Motility was measured for two hours using WMicrotracker machine (InVivo Biosystems, Eugene, OR, USA) with preset configurations.

### 2.5. RNA Sequencing

Animals were synchronized by bleaching and plated as L1 larvae on control or protein powder 2-containing 10 cm NGM agar plates seeded with *E. coli* OP50. At the L4 larval stage, animals were transferred to 10 cm NGM agar plates containing 5-FU (10 µM) (Merck, #F6627) to prevent progeny production. Animals were collected on day 2 of adulthood (three biological replicates for both strains) and frozen in liquid nitrogen. Total RNA was extracted with TRIzol Reagent (ThermoFisher Scientific, Waltham, MA, USA), #15596018). Samples were sent to Novogene for library construction, quality control, sequencing, and data analysis. In short, mRNA was purified from total RNA using poly-T oligo-attached magnetic beads. After the fragmentation, the first strand cDNA was synthesized using random hexamer primers, followed by the second strand cDNA synthesis. The library preparations were sequenced on an Illumina platform. Paired-end clean reads were mapped to the reference genome using HISAT2 software (version 2.0.5) [16]. FeatureCounts [17] was used to count the read numbers mapped of each gene. Differential expression analysis between two conditions (three biological replicates per condition) was performed using DESeq2 [18]. Genes with *p*-value < 0.05 and log2FoldChange > 0 found by DESeq2 were assigned as differentially expressed. Differentially expressed genes can be found from Supplementary Materials File S2. GO enrichment analysis was performed using clusterProfiler [19]. The RNA-seq data are available in the Gene Expression Omnibus (GEO) database repository (GSE270138).

### 2.6. Quantitative RT-PCR (qRT-PCR)

Animals were synchronized by bleaching and plated as L1 larvae on 10 cm NGM agar plates. Plates were kept at 20 °C. Animals were collected at the L4 larval stage or on day 2 of adulthood and frozen in liquid nitrogen. Animals that were collected on day 2 of adulthood were transferred to plates containing 5-FU (10 µM) (Merck, #F6627) at the L4 larval stage to prevent progeny production. TRIzol Reagent (ThermoFisher Scientific, #15596018) was used to extract RNA. cDNA synthesis was performed with the QuantiTect Reverse Transcription Kit (Qiagen, Hilden, Germany), #205313), and qRT-PCR reactions were run with the HOT FIREPol SolisGreen qPCR Mix reagent (Solis BioDyne, Tartu, Estonia), #08-46-00001) using the CFX384 or CFX Opus 384 machine (Bio-Rad, Hercules, CA, USA). qRT-PCR data were normalized to the expression of *cdc-42* and *pmp-3*. qRT-PCR oligos used in this study are provided in Supplementary Materials File S1, Table S2. qRT-PCR experiments were performed with three biological replicates, with three technical replicates for each biological replicate. All qRT-PCR experiments were performed at least twice.

### 2.7. Pseudomonas aeruginosa Assay

*Pseudomonas aeruginosa* assays were performed as described earlier [20] with minor modifications. A total volume of 200 μL of MProPow solutions (1 mg/mL) was added to 3 cm NG plates with 10 µM 5-FU (Merck, #F6627). After the plates had dried, they were seeded with 3 µL of an overnight-grown *Pseudomonas aeruginosa* (PA14) suspension and incubated at 37 °C for 24 h. A total volume of 20 µL of 2% SDS was added to the edges of the plate to prevent the escape of the animals. *C. elegans* were grown on 6 cm NGM agar plates supplemented with 1 mL of MProPow solutions (1 mg/mL) and seeded with OP50. Animals were transferred to PA14 plates at the L4 stage and incubated at 25 °C. Animals were scored daily for survival based on their ability to respond to touch. Animals that crawled off the plate were censored. Mean lifespan on PA14 ± standard error (SE) is reported in the Supplementary Materials File S1, Table S1.

### 2.8. RNA Interference (RNAi)

*cpr-3* RNAi clone was taken from the Ahringer RNAi library (Source BioScience, Nottingham, UK). RNAi was performed using the feeding protocol described earlier [15]. Animals were kept on *cpr-3* RNAi during the whole experiment.

### 2.9. Statistical Analysis

Statistical analyses for motility and qRT-PCR data were carried out in GraphPad Prism (version 10.2.3). qRT-PCR data represent the mean of three biological replicates ± standard deviation (SD). One-way ANOVA was used for qRT-PCR and motility measurements to analyze whether there are any statistically significant differences in gene expression or motility, respectively, between the means of three or more independent groups (e.g., Ctrl, MProPow1 and MProPow2). In Figure S1b, *t*-test was used to determine the significance of the difference between the means of two sets of data. In all statistical tests, a *p*-value less than 0.05 was considered significant. Statistical calculations for lifespan experiments were carried out in RStudio (version 1.1.463) using the Cox-proportional hazard regression analysis. Statistical details for the lifespan data can be found in Supplementary Materials File S1, Table S1.

## 3. Results

### 3.1. MFGM-Containing Protein Powder (MProPow) Improves C. elegans Motility

In this study, we used two MFGM-containing protein powders (MProPows, MProPow1, and MProPow2), which were generated using the same recipe but are from two independent batches (see Methods for information on MProPow production and Table 1 for its nutritional content). Regarding the concentration of MProPows used in the experiments, it has been shown that single amino acids extend *C. elegans* lifespan in liquid S-medium when supplemented at concentrations of 1–10 mM [21]; 10 mM of serine, which shows the most prominent effect on lifespan [21], equals approximately a concentration of 1 mg/mL. Since a single amino acid has a robust effect on lifespan at this concentration, we decided to investigate whether MProPows affect physiology at similar concentrations. For this, we mixed MProPows with water to achieve a final concentration of 1 mg/mL and spread the solutions on NGM agar plates (see Methods for details).

**Figure 1:**
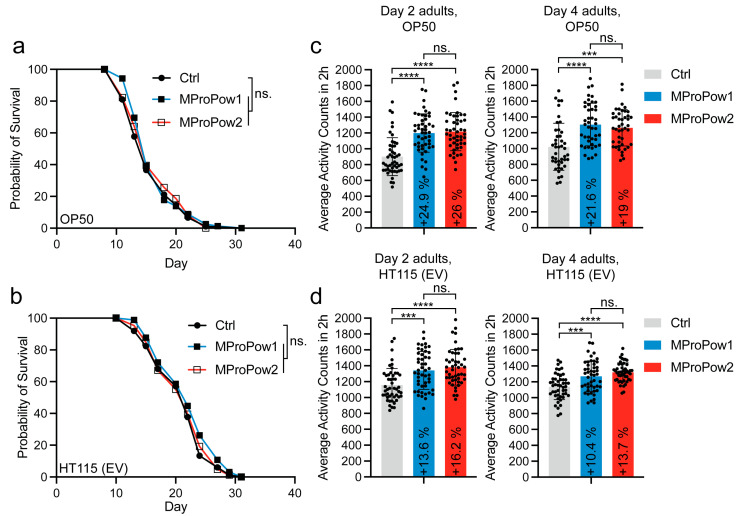
MProPows do not affect *C. elegans* lifespan, but enhance motility. (**a**) Lifespan of OP50-fed and (**b**) HT115 (EV)-fed *C. elegans* on plates supplemented with MProPow1 or MProPow2. Lifespan statistics are reported in Supplementary Materials File S1, Table S1. (**c**) Motility of day 2 and day 4 adult (day 5 and day 7 from hatch, respectively), OP50-fed and (**d**) HT115 (EV)-fed *C. elegans* on plates supplemented with MProPow1 or MProPow2. Each dot (one well in a 96-well plate) represents activity counts for a group of 10 animals over two hours (n = 440 animals for day 4 adults on OP50, n = 480 animals for other conditions). Data are combined from two independent experiments (*** *p* < 0.001, **** *p* < 0.0001, ns: not significant, one-way ANOVA with Tukey’s test).

First, we tested whether MProPows affect lifespan. For lifespan experiments, we used the *E. coli* B strain OP50, which is probably the most commonly used bacterial strain in *C. elegans* maintenance and experiments, as a food source. Additionally, we utilized the *E. coli* K-12 strain HT115, another commonly used bacterial strain in *C. elegans* research, particularly in RNAi experiments. In this experiment, we employed HT115 bearing the empty vector (EV), which serves as a control in RNAi experiments. Notably, HT115 provides a healthier diet compared to OP50, as it not only improves the host’s response to oxidative, heat, or pathogenic stress [22] but also leads to a longer lifespan [23,24,25]. In the lifespan assays performed at 20 °C, we found that MProPows do not affect lifespan on either diet (Figure 1a,b, Supplementary Materials File S1, Table S1). In addition to the lifespan experiments conducted at 20 °C, we also tested the effects of MProPows on the lifespan of animals kept at 25 °C, a condition that induces mild heat stress. We conducted two independent experiments at this higher temperature. One experiment showed no statistical difference between the treatments, while the other experiment indicated that MProPows induce a modest lifespan extension (Figure S1a, Supplementary Materials File S1, Table S1). Nevertheless, based on our data, it can be concluded that MProPows do not affect lifespan.

Importantly, lifespan does not always correlate with healthspan, and therefore, many interventions that prolong lifespan also extend the period of frailty [26,27]. To investigate whether MProPows affect organismal health, we used a thrashing assay in liquid, which measures *C. elegans* motility and is a widely used method to analyze the fitness of the animals [26,27]. In this assay, we placed animals in M9 solution and measured their swimming activity on a 96-well plate using WMicrotracker (see Methods for details). Interestingly, when analyzing day 2 and day 4 adult animals (day 5 and day 7 from hatch, respectively) grown on OP50, we found that both MProPow1 and MProPow2 increase the motility of the animals (Figure 1c). MProPows also increase the motility of animals grown on HT115 (Figure 1d), although their effect is not as pronounced as in experiments performed with OP50 (Figure 1c). Importantly, the HT115 diet alone leads to elevated motility compared to OP50-fed animals (Figure S1b), which may explain the finding that MProPows have a greater effect on animals maintained on OP50. Nevertheless, these data demonstrate that, although MProPows do not affect lifespan, they promote the fitness of the animals.

### 3.2. MProPow Decreases the Expression of Genes Related to Innate Immunity, and Reduces the Survival on Pathogenic Bacteria

**Figure 2:**
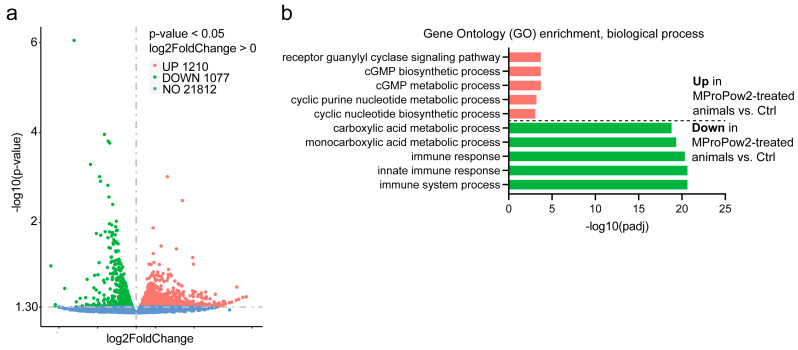
RNA sequencing of MProPow2-treated animals. (**a**) Volcano plot showing differentially expressed genes in of OP50-fed *C. elegans* treated with MProPow2 compared to control. (**b**) Enriched GO terms among up- and downregulated genes in MProPow2-treated animals. Lists of differentially expressed genes are shown in Supplementary Materials File S2.

To elucidate the mechanism by which MProPows promote fitness, we performed RNA-seq of OP50-fed day 2 adult *C. elegans* grown on MProPow2. Data analysis revealed that the expression of 1210 genes is upregulated, and 1077 genes are downregulated in MProPow2-treated animals (Figure 2a, Supplementary Materials File S2). Interestingly, Gene Ontology (GO) enrichment analysis of differentially expressed genes revealed that the downregulation of innate immunity-related processes shows the most significant enrichment in MProPow2-treated animals (Figure 2b, Supplementary Materials File S2). Although the GO terms “monocarboxylic acid metabolic process” and “carboxylic acid metabolic process” also show strong enrichment among downregulated genes in MProPow2-treated animals (Figure 2b), the downregulation of innate immunity-related genes in three biological replicates is statistically more significant compared to the genes falling under the two above-mentioned GO terms. For example, of the 22 most significantly downregulated genes, eight are under the innate immunity-related GO terms (Supplementary Materials File S2). In contrast, of the 106 most significantly downregulated genes, only three and four genes fall under the GO terms of “monocarboxylic acid metabolic process” and “carboxylic acid metabolic process”, respectively (Supplementary Materials File S2).

**Figure 3:**
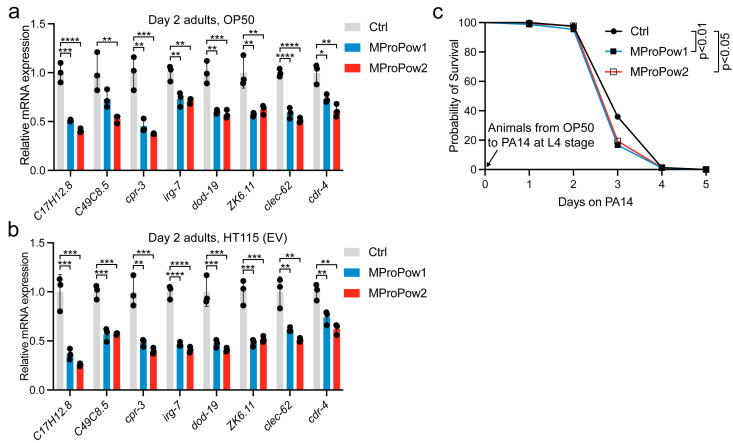
MProPows reduce the expression of genes related to innate immunity. (**a**) qRT-PCR of selected innate immunity-related genes in OP50-fed and (**b**) HT115 (EV)-fed day 2 adult *C. elegans* grown on control or MProPow-supplemented plates. Bars represent mRNA levels relative to control with error bars indicating mean ± SD of three biological replicates, each with three technical replicates (* *p* < 0.05, ** *p* < 0.01, *** *p* < 0.001, **** *p* < 0.0001, one-way ANOVA with Tukey’s test). (**c**) Survival of control and MProPow-treated animals on *Pseudomonas aeruginosa* (PA14). Statistics for PA14 assays are reported in Supplementary Materials File S1, Table S1.

Due to the downregulation of innate immunity-related genes in MProPow2-treated animals, we focused on this biological process. To validate the RNA-seq data, we performed qRT-PCR analysis on the eight most significantly downregulated innate immunity-related genes from independent samples, including animals treated with MProPow1. These experiments revealed that both MProPows reduce the expression of innate immunity genes in day 2 adult animals (Figure 3a). Based on these data, we hypothesized that MProPow-treated animals might have an upregulated immune response earlier in development, which is then suppressed in post-developmental life stages. Therefore, we examined the expression of innate immunity genes at the L4 larval stage (day 3 from hatch, the last larval stage before adulthood). Contrary to our expectations, most of the examined genes showed reduced expression in L4 larvae as well (Figure S1c). In addition to OP50, we tested whether MProPows affect the expression of innate immunity-related genes in animals fed with HT115 (EV) and found that MProPows also diminish the expression of innate immunity genes in day 2 adults on this diet (Figure 3b). Together, these data demonstrate that MProPows regulate the expression of innate immunity-related genes in a diet-independent manner. Moreover, as the bacterial diet forms the *C. elegans* gut microbiome, our results demonstrate that MProPows affect host physiology independently of these commensal bacteria.

As MProPows reduce the expression of genes functioning in innate immunity (Figure 2b and Figure 3a,b), one obvious question arises: do they impair survival on pathogenic bacteria? To investigate this, we performed a slow-killing assay with Pseudomonas aeruginosa strain PA14 [20]. Of the three independent experiments, one did not show differences in survival between control and MProPow-treated animals (Supplementary Materials File S1, Table S1). However, in the two other experiments, MProPows reduced survival on PA14 (Figure 3c, Supplementary Materials File S1, Table S1). These data suggest that, reflecting the decreased expression of genes associated with innate immunity, MProPows may slightly suppress the innate immune response against pathogenic bacteria.

### 3.3. Downregulation of cpr-3 Promotes Fitness

**Figure 4:**
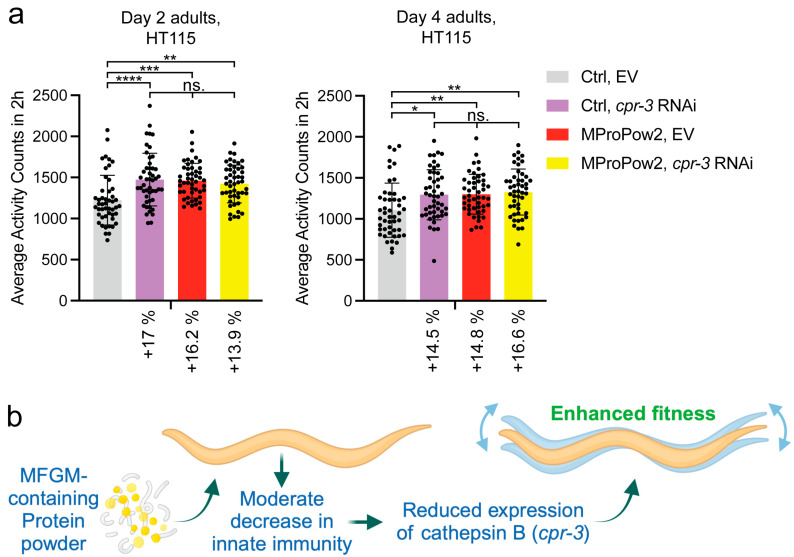
Knockdown of MProPow-regulated cathepsin B enhances motility. (**a**) Motility of day 2 and day 4 adult, control (EV) or *cpr-3* RNAi-treated animals grown on control or MProPow2-supplemented plates. Each dot (one well in a 96-well plate) represents activity counts for a group of 10 animals over two hours (n = 480 animals for all conditions). Data are combined from two independent experiments (* *p* < 0.05, ** *p* < 0.01, *** *p* < 0.001, **** *p* < 0.0001, ns: not significant, one-way ANOVA with Tukey’s test). (**b**) Model based on data presented in this study. Illustration was created with BioRender.com.

Since MProPows decrease survival on pathogenic bacteria (Figure 3c, Supplementary Materials File S1, Table S1), we hypothesized that this is a trade-off for enhanced fitness (Figure 1c,d). In other words, we asked whether the downregulation of innate immunity-related genes promotes fitness. Notably, one of the most significantly downregulated genes in MProPow-treated animals is *cpr-3* (Figure 3a,b and Figure S1c, Supplementary Materials File S2). CPR-3 is a homolog of cathepsin B (CTSB), a lysosomal cysteine protease [28]. Importantly, cathepsin B has been associated with many pathologies [29]. For example, clinical findings from multiple studies have shown that CTSB levels are increased in many neurologic conditions, including several neurodegenerative diseases such as Alzheimer’s disease and traumatic brain injury [30]. Similarly, as in humans, CTSB levels are increased in animals modeling neurologic disorders, and as shown in 12 studies, its deletion leads to significant improvements in behavioral deficits and neuropathology in these animal models [30]. Furthermore, CTSB plays a role in the development of cancer as well as in conditions such as lung and cardiovascular disorders [31]. Based on these reports, we examined how *cpr-3* RNAi affects *C. elegans* fitness. Strikingly, we found that *cpr-3* knockdown leads to a significant increase in the motility of both day 2 and day 4 adult *C. elegans* (Figure 4a). Notably, *cpr-3* RNAi does not further increase the motility of MProPow2-treated animals (Figure 4a), indicating that these interventions promote fitness through the same mechanism.

## 4. Discussion

We show here that MFGM-containing protein powder (MProPow) promotes fitness, which is, at least partly, due to the downregulation of *cpr-3* expression (Figure 4b). Interestingly, MProPow uncouples fitness from lifespan, as it does not affect longevity (Figure 1a,b). These data indicate that its effect on fitness (Figure 1c,d) is not mediated through known lifespan-modulating mechanisms. Furthermore, the finding that MProPow enhances fitness on both *E. coli* OP50 and HT115 is important, as it indicates that the effect is independent of both diet and gut microbiome. To highlight the influence of diet/microbiome in *C. elegans* health and lifespan experiments, metformin, a widely used drug for type 2 diabetes, has been shown to promote longevity on OP50 but not on the healthier HT115 strain [32]. In this respect, considering that MFGM may be beneficial in the treatment of obesity and the associated type 2 diabetes [33,34], MProPow could be additive to the effects of metformin in these conditions.

With regard to the finding that *cpr-3* RNAi increases motility (Figure 4a), one might think that the phenotype is due to downregulation of CPR-3 function in muscle cells. However, data from a fluorescent reporter strain have shown that *cpr-3* is expressed in the pharynx, pharyngeal–intestinal valve, intestine, and rectal gland cells, with the intestine showing the strongest signal [35]. Therefore, in our experiments, it is likely that the MProPow-induced reduction in *cpr-3* expression in the intestine promotes organismal fitness. This leads to the question: could a similar mechanism apply to humans? In humans, CTSB is not only ubiquitously expressed [36] (Human Protein Atlas, proteinatlas.org), but also secreted. Interestingly, running-induced systemic CTSB secretion from muscles induce beneficial cognitive effects [37], whereas CTSB secretion from many other cell types can have aggravating effects (for example, through the modulation of extracellular matrix (ECM) [38]), especially under pathological conditions [31,39,40]. Thus, together with the physiological condition, the source tissue may affect whether the secreted CTSB is beneficial or not. Notably, adipose tissue has been shown to be one of the tissues that secrete CTSB [41]. Given that the intestine, which expresses *cpr-3* at high level [35], is the primary adipose tissue in *C. elegans* [42], one intriguing hypothesis is that intestinal cells secrete CPR-3, which then modulates the ECM in, for example, muscle cells. Adipose tissue-secreted CTSB could have the same function in humans. In this respect, adipose tissue has been associated with the regulation of muscle function, as aging-associated adipose inflammation in obese people can lead to fatty infiltration in skeletal muscles, resulting in decreased muscle strength and functionality [43]. Nevertheless, further research is required to elucidate whether MProPow modulates CTSB levels in humans.

In addition to *cpr-3*, MProPow was also found to reduce the expression of many other genes related to innate immunity (Figure 2, Figure 3a,b and Figure S1c, Supplementary Materials File S2). Consistent with the gene expression data, MProPow moderately reduces survival on pathogenic PA14 (Figure 3c). We hypothesized above that this decrease in innate immunity is a trade-off for enhanced fitness (Figure 1c,d). Supporting this hypothesis, it has been shown that the allocation of energy toward immune function restricts physical growth in children and preadolescents [44,45]. Additionally, a meta-analysis of data from poultry suggests that organisms have to make a trade-off between immune function and other fitness-enhancing traits [46]. Moreover, the immune response restricts plant development, and aberrantly activated innate immunity is toxic to *C. elegans* [47], further underlining the conservation of the trade-off between immunity and fitness.

Our observed effect of MProPow on *C. elegans* immunity is somewhat contradictory to findings in humans, as numerous studies have reported that MFGM enhances immunity in infants [6,48], whereas milk-derived peptides have also shown promise as immunity-enhancing molecules [49]. In addition to the trade-off hypothesis presented above, there are two other possible explanations. Firstly, *C. elegans* and humans diverged during evolution, and unlike humans, *C. elegans* has not developed adaptive immunity. Therefore, this model organism cannot be used to study the effects of MProPow on this specific branch of the immune system. Secondly, since we assessed the survival of *C. elegans* on PA14 at the adult stage, and considering that MFGM modulates the immunity of newborns [6,48], one explanatory factor for the opposing results could be the developmental stage when exposed to pathogens. On the other hand, milk-derived peptides and MFGM have also been shown to promote health by suppressing the production of multiple inflammation-associated proteins such as IL-1β, IL-6, and TNF-α (suppressed by MFGM [50,51]), as well as MCP-1 (suppressed by milk-derived peptides [52]), which raises the possibility that MProPow-mediated suppression of the immune response is beneficial in humans. Interestingly, inhibition of CTSB leads to a reduced expression of all four aforementioned inflammatory gene [53]. Hence, considering that MProPow reduces *cpr-3*/*CTSB* expression in *C. elegans* (Figure 3a,b and Figure S1c), it is possible that milk-derived peptides and MFGM modulate immunity through CTSB in mammals.

Finally, the data presented here, which show that MProPow promotes *C. elegans* fitness, support the findings from a clinical study performed earlier [9]. Although these two studies were performed in organisms that diverged in evolution hundreds of millions of years ago, similar results from both systems provide strong evidence that the tested MProPow possesses beneficial biological activity. Referring to the effect of MProPow on cathepsin B (*cpr-3*) expression (Figure 3a,b and Figure S1c), the efficacy of almost twenty CTSB inhibitors has been tested in the treatment of various diseases, ranging from cancer to nervous system-associated maladies [29]. Thus, if MProPow is found to decrease CTSB mRNA levels in humans, it would provide a complementary mechanism to target CTSB activity in disease.

## 5. Conclusions

In this study, we found that MFGM-containing protein powder (MProPow) does not affect lifespan but enhances fitness in *C. elegans*. Additionally, MProPow was found to reduce the expression of innate immunity-associated genes, consequently impairing survival on pathogenic bacteria. Interestingly, one of the innate immunity-associated genes downregulated by MProPow is *cpr-3*, the homolog of cathepsin B (CTSB). We show that RNAi of *cpr-3* enhances fitness, but not in MProPow-treated animals, suggesting that the positive effects of MProPow on animal physiology can at least partly be explained by the downregulation of *cpr-3*/*CTSB* expression. In conclusion, this study validates *C. elegans* as a model organism for the preliminary screening of nutraceuticals, allowing for a cost-effective and relatively easy assessment of the effectiveness of bioactive components. Furthermore, it highlights the delicate balance between different aspects of health in biological systems (the trade-off between immunity and fitness) and provides a scientific basis for the development of milk-based supplements and food products aimed at improving physical performance and overall fitness.

## Figures and Tables

**Table 1:** Nutritional content of MProPow. Table is modified from Jyväkorpi et al. [9].

Nutritional Content	Amount per 100 g of MProPow [9]
Energy, kcal	467
Protein, g	76.7
Fat, g	13.3
Carbohydrates, g	6.7
Lactose, g	0.0
MFGM, g	13.0
Phospholipids, g	4.3

## Data Availability

The RNA-seq data generated during the current study are available in the Gene Expression Omnibus (GEO) database repository (GSE270138).

## Supplementary Materials

The following supporting information can be downloaded at: https://www.mdpi.com/article/10.3390/nu16142290/s1, Supplementary Materials File S1: Figure S1: Lifespan at 25 °C, motility on *E. coli* OP50 and HT115 (EV), and the expression of innate immunity genes in L4 larvae; Table S1: Individual replicates of *C. elegans* lifespan experiments; Table S2: Oligonucleotide sequences used in qRT-PCR; Supplementary Materials File S2: Differentially expressed genes.
