# Supplementary material for: Milk Fat Globule Membrane-Containing Protein Powder Promotes Fitness in Caenorhabditis elegans
Source: Nutrients. 2024 Jul 17;16(14):2290. doi: 10.3390/nu16142290 (PMC11280102; doi:10.3390/nu16142290)
Supplement: Supplementary file 1 [file nutrients-16-02290-s001.zip › Supplementary Materials file S1 .pdf]

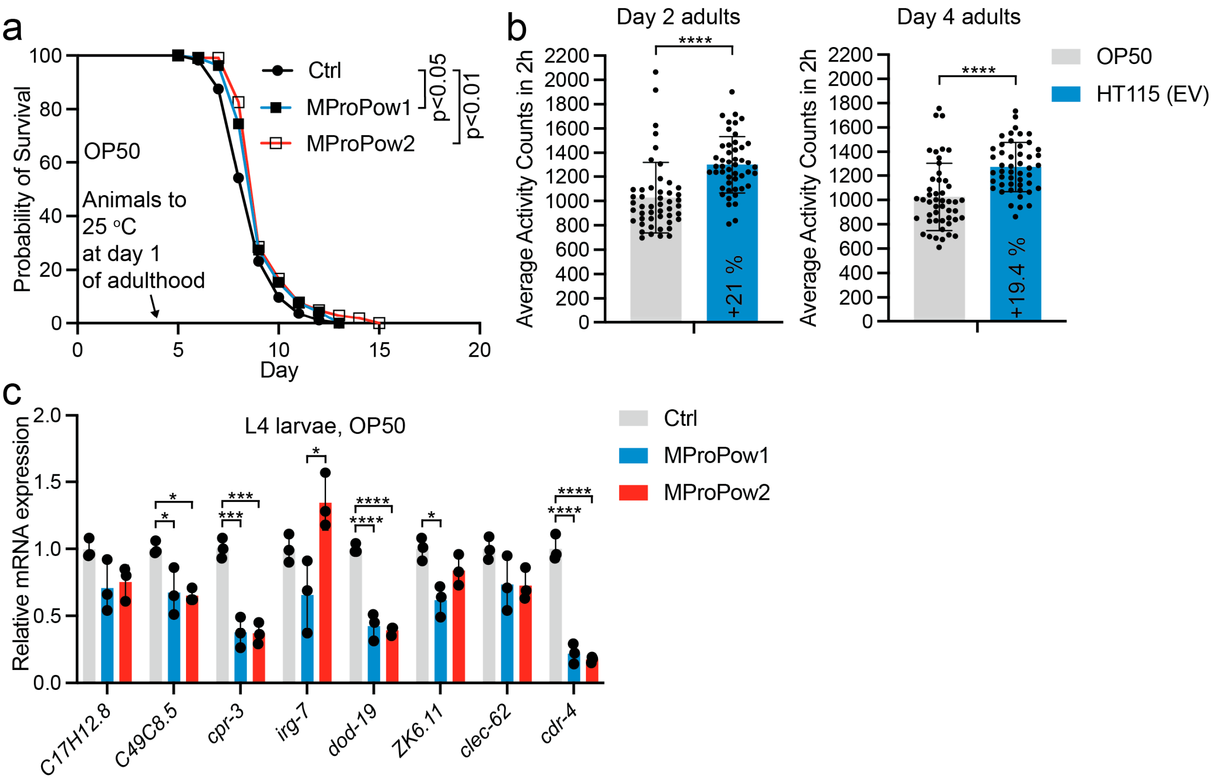

Figure S1. Lifespan at 25 °C, motility on *E. coli* OP50 and HT115 (EV), and the expression of innate immunity genes in L4 larvae. (**a**) Survival of OP50-fed *C. elegans* on control, MProPow1- and MProPow2-supplemented plates. Animals were transferred from 20°C to 25°C at day 1 of adulthood (4th day from hatch). Lifespan statistics are reported in Supplementary Information file 1, Table S1. (**b**) Motility of OP50- and HT115 (EV)-fed *C. elegans* on day 2 and day 4 of adulthood (day 5 and day 7 from hatch, respectively). Each dot (one well in a 96-well plate**)** represents activity counts for a group of 10 animals over two hours (n = 480 animals for all conditions). Data are combined from two independent experiments (****p < 0.0001, t-test). (**c**) qRT-PCR of selected innate immunity-related genes in OP50-fed, L4 larval stage (day 3 from hatch) *C. elegans elegans* grown on control or MProPow-supplemented plates. Bars represent mRNA levels relative to control with error bars indicating mean ± SD of three biological replicates, each with three technical replicates (*p < 0.05, ***p < 0.001, ****p < 0.0001, one-way ANOVA with Tukey's test).

Table S1. Individual replicates of *C. elegans* lifespan experiments

Genotype and treatment	mean lifespan ± SE (days)	variation compared to control (%)	*P*-values against control	N
**Figure 1a**				
N2 (OP50) Ctrl	15.9 ± 0.45			91
N2 (OP50), MProPow1	16.5 ± 0.45	+ 3.6	0.414	81
N2 (OP50), MProPow2	16.2 ± 0.45	+ 1.9	0.621	88
**Figure 1a**				
N2 (OP50) Ctrl	15.3 ± 0.44			85
N2 (OP50), MProPow1	16.8 ± 0.41	+ 8.9	0.0807	67
**Figure 1a**				
N2 (OP50) Ctrl	16.5 ± 0.47			90
N2 (OP50), MProPow2	17.2 ± 0.54	+ 4.1	0.357	73
**Figure 1a**				
N2 (OP50) Ctrl	14.8 ± 0.39			
N2 (OP50), MProPow2	15.8 ± 0.57	+ 6.3		
**Figure 1b**				
N2 (HT115, EV) Ctrl	20.8 ± 0.48			85
N2 (HT115, EV) MProPow1	20.8 ± 0.44	+ 0	0.673	85
N2 (HT115, EV) MProPow2	21.5 ± 0.53	+ 3.3	0.492	68

**Figure 1b**				
N2 (HT115, EV) Ctrl	21.0 ± 0.49			83
N2 (HT115, EV) MProPow1	22.0 ± 0.56	+ 4.5	0.135	67
N2 (HT115, EV) MProPow2	21.2 ± 0.49	+ 0.9	0.765	84
**Figure 1b**				
N2 (HT115, EV) Ctrl	20.3 ± 0.55			85
N2 (HT115, EV), MProPow2	21.4 ± 0.55	+ 5.1	0.614	68
**Figure S1a**				
N2 (OP50) Ctrl, 25 ºC from day1 of adulthood	8.78 ± 0.13			92
N2 (OP50) MProPow1, 25 ºC from day1 of adulthood	9.25 ± 0.14	+ 5.1	0.040294	93
N2 (OP50) MProPow2, 25 ºC from day1 of adulthood	9.44 ± 0.14	+ 7	0.003277	102
**Figure S1a**				
N2 (OP50) Ctrl, 25 ºC from day1 of adulthood	9.66 ± 0.14			94
N2 (OP50) MProPow1, 25 ºC from day1 of adulthood	9.96 ± 0.17	+ 3	0.1824	98
N2 (OP50) MProPow2, 25 ºC from day1 of adulthood	10.08 ± 0.16	+ 4.2	0.0688	106
**Figure 3c (days on PA14)**				
N2 (PA14) Ctrl	3.02 ± 0.029			155
N2 (PA14) MProPow1	3.03 ± 0.032	+ 0.3	0.718	156
N2 (PA14) MProPow2	2.95 ± 0.038	- 2.3	0.725	155

Figure 3c (days on PA14)				
N2 (PA14) Ctrl	3.35 ± 0.051			103
N2 (PA14) MProPow1	3.12 ± 0.046	- 6.9	0.004041	117
N2 (PA14) MProPow2	3.1 ± 0.028	- 7.5	0.021594	115

Figure 3c (days on PA14)				
N2 (PA14) Ctrl	3.50 ± 0.068			134
N2 (PA14) MProPow1	3.23 ± 0.046	- 7.7	0.000319	143
N2 (PA14) MProPow2	3.17 ± 0.042	- 9.4	8.84e-06	146

Table S2. Oligonucleotide sequences used in qRT-PCR

Gene	Forward (5’ → 3’)	Reverse (5’ → 3’)
cdc-42	CTGCTGGACAGGAAGATTACG	CTCGGACATTCTCGAATGAAG
pmp-3	GTTCCCGTGTTCATCACTCAT	ACACCGTCGAGAAGCTGTAGA
C17H12.8	CACTGTCGATTGCTCACTCC	TCCGGTGCTGATGTATCCTT
C49C8.5	CGACTCAACTCTGCTTCTTGG	AGTGAAAAGAGCCATTCGAACT
cpr-3	GTGATTTCCGACCGAGTGTG	CTCCACCTGTTACTGCTCCA
irg-7	CGTGCCGGAACTATGTTCTG	ATCTCCGCTAGCTGTTCCTC
dod-19	AGTACCTCAGCCGACACTTC	AGCATCATCGAAATTGTAACGGA
ZK6.11	TGGCATATCTGTACGCTGGT	TGCTACCAAGGTCAACGAGA
clec-62	GCAAGAACAACACTCGCAAA	TGCTAACACCAGACGCCTTA
cdr-4	CGCTTCTGACTCGCTTTACA	TGCTCCAACACATCGGTAGT
